# A new model for solving stochastic second-order cone complementarity problem and its convergence analysis

**DOI:** 10.1186/s13660-018-1814-8

**Published:** 2018-08-29

**Authors:** Meiju Luo, Caihua Zhang

**Affiliations:** School of Mathematics, Liaoning University, Liaoning, China

**Keywords:** 90C33, 90C15, 65K10, Second-Order cone complementarity problem, Conditional value-at-risk, Sample average approximation, Smoothing function, Convergence, Level set

## Abstract

In this paper, we mainly consider the stochastic second-order cone complementarity problem (SSOCCP). Due to the existence of stochastic variable, the SSOCCP may have no solutions. In order to deal with this problem, we first regard the merit function of the stochastic second-order cone complementarity problem as the loss function and then present a low-risk deterministic model that is a conditional value-at-risk (CVaR) model. However, there may be two difficulties for solving the CVaR model directly: One is that the objective function is a non-smoothing function. The other is that the objective function contains expectation. (In general, the value of expectation is not easy to be calculated.) In view of these two problems, we present the approximation problems of the model by using a smoothing method and a sample average approximation technique. Furthermore, we give the convergence results of global optimal solutions and the convergence results of stationary points of the approximation problems, respectively.

## Introduction

The second-order cone in $R^{n}$ is defined as

$$\begin{aligned} \mathcal{K}^{n} = \bigl\{ (x_{1},x_{2})\in R\times R^{n-1}| \Vert x_{2} \Vert \leq x_{1} \bigr\} , \end{aligned}$$

where $\|\cdot\|$ denotes the Euclid norm.

Second-order cone complementarity problem (SOCCP) is as follows: Find a vector $x\in R^{n}$ satisfying

$$\begin{aligned} x\in\mathcal{K}, f(x)\in\mathcal{K}, \quad x^{T}f(x)=0, \end{aligned}$$

where

1.1 $$\begin{aligned} \mathcal{K}=\mathcal{K}^{n_{1}}\times\cdots\times \mathcal{K}^{n_{m}}, \end{aligned}$$

and $n_{1}+\cdots+n_{m}=n$.

The KKT condition of second-order cone programming (SOCP) problem can be equivalent to a second-order cone complementarity problem. So, many researchers pay attention to considering SOCCP.

Recently, the researchers have obtained many good achievements about SOCCP. Many researchers have proposed some methods for solving SOCCP. For example, Alizadeh et al. and Andersen et al. gave the interior point method [1, 2]. Fukushima et al. gave the smoothing method [10]. Chen et al. gave the nonsmoothing method [3, 5]. Especially, Fukushima et al. [10] proved that the min function and the FB function in NCP can be spread to SOCCP by using Jordan algebra. They constructed a smoothing function of natural residual function and gave some properties of the smoothing function’s Jacobian matrix. In fact, many practical problems can be transformed into SOCCP. For example, Kanno et al. gave three-dimensional quasi-frictional contact by using second-order cone linear complementarity [12] and Hayashi et al. gave robust Nash equilibria [11]. However, there are many stochastic factors such as price, supply, and demand in practice. So, in this paper, we mainly consider SOCCP with stochastic factors, that is, stochastic second-order cone complementarity problem (SSOCCP). SSOCCP is to find a vector $x\in R^{n}$ such that

1.2 $$\begin{aligned} x\in\mathcal{K}, f(x,\omega)\in\mathcal{K},\quad x^{T}f(x,\omega )=0, \quad\omega\in\Omega, \mbox{ a.s.}, \end{aligned}$$

where $\mathcal{K}$ is given by (1.1). $\omega\in\Omega$ is a stochastic variable which is defined in the probability space $(\Omega ,\mathcal{F},\mathcal{P})$, $f:R^{n}\times\Omega\rightarrow R^{n}$ is a continuously differentiable function about *x*. “a.s.” is the abbreviation of “almost surely”. Because of the existence of stochastic factor *ω*, generally there is no vector *x* satisfying (1.2) for all $\omega\in \Omega$. In order to give reasonable solutions of (1.2), we present a low risk deterministic model and regard the solutions of this model as the solutions of SSOCCP.

For simplicity, we assume that $\mathcal{K}=\mathcal{K}^{n}$. This assumption has not lost generality, the results of this research can be easily extended to the general situation. In this paper, we assume that $\Omega\subset R^{m} $ is a nonempty and compact set. $f(x,\omega)$ is a twice continuously differentiable function with respect to *x*, and $f(x,\omega)$ is a continuously differentiable function with respect to $\omega\in\Omega$. $\rho(\cdot)$ is a continuity probability density function. For convenience, we use $x=(x_{1},x_{2} )$ to denote $(x_{1}^{T},x_{2}^{T})^{T}$, $\nabla g(x)$ to denote gradient of $g:R^{n}\rightarrow R$ with respect to *x*. And $R_{+}:=\{x\in R\mid x\geq0\}$. $A\in R^{m\times n}$ is a matrix, while $\|A\|$ denotes the spectral norm. $\|A\|_{\mathcal{F}}$ denotes the Frobenius norm, which is defined as

$$\begin{aligned} \Vert A \Vert _{\mathcal{F}}:=\sqrt{\sum _{i=1}^{n}\sum_{j=1}^{n} \vert a_{ij} \vert ^{2}}. \end{aligned}$$

The rest of our paper is organized as follows. In Sect. 2, we give some preliminaries, including second-order cone complementarity function, *τ*-$R_{0}$ function, and stochastic *τ*-$R_{0}$ function. In Sect. 3, based on CVaR, we give a deterministic model for solving SSOCCP. Then we use the sample average approximation and smoothing method to solve this model. In Sect. 4, when $f(x,\omega)$ is a stochastic *τ*-$R_{0}$ function, we consider the boundedness of the level set. In Sect. 5, we consider the convergence of a global optimal solutions sequence and a stationary points sequence of the model which is referred to in Sect. 3. Conclusions are given in Sect. 6.

## Preliminaries

In this section, we first describe some concepts and properties from Euclidean Jordan algebras that are needed in this paper. All these can be found in the book [8].

An Euclidean Jordan algebra is a triple $(\textsl{V},\circ,(\cdot,\cdot ))$, where $(\textsl{V},(\cdot,\cdot))$ is a finite dimensional inner product space over *R* and $(x,y): \textsl{V}\times\textsl{V}\rightarrow \textsl{V}$ is a bilinear mapping satisfying the following conditions:

$$\begin{aligned}& (1)\quad x\circ y=y\circ x \quad\mbox{for all } x,y\in\textsl{V}, \\& (2)\quad x\circ \bigl(x^{2}\circ y \bigr)=x^{2}\circ(x \circ y) \quad\mbox{for all }x,y\in\textsl{V},\mbox{ where } x^{2}:=x \circ x, \\& (3)\quad (x\circ y,z)=(y,x\circ z)\quad\mbox{for all } x,y,z\in\textsl{V}. \end{aligned}$$

In addition, there is an element $e\in\textsl{V}$ (called the unit element) such that $x\circ e=x$ for all $x\in\textsl{V}$. More specifically, for any $x=(x_{1},x_{2})\in R\times R^{n-1}$, $y=(y_{1},y_{2})\in R\times R^{n-1}$, their Jordan product associated with $\mathcal{K}^{n}$ is defined as

$$\begin{aligned} x\circ y= \bigl(x^{T}y,y_{1}x_{2}+x_{1}y_{2} \bigr). \end{aligned}$$

Usually, we use $x+y$ to denote the sum of the corresponding components, that is, $x+y=(x_{1}+y_{1},x_{2}+y_{2})$. Moreover, $x^{2}$ denotes $x\circ x$ and $\sqrt{x}$ denotes a vector, which satisfies $\sqrt{x}\circ\sqrt{x}=x$.

### Theorem 2.1

([8], The spectral decomposition theorem)

*Let* *V* *be an Euclidean Jordan algebra*. *Then there is a number* *r* *such that*, *for every* $x\in\textsl{V}$, *there exist a Jordan frame* $\{e_{1}, e_{2},\ldots, e_{r}\}$ *and real numbers* $\lambda_{1}, \lambda _{2},\ldots, \lambda_{r}$ *with* $x=\lambda_{1}e_{1}+\cdots\lambda _{r}e_{r}$. *Here*, $e_{i}\circ e_{j}=0$ *if* $i\neq j$ *and* $\sum_{i=1}^{r}e_{i}=e$, *the numbers* $\lambda_{i}$ ($i=1,2,\ldots,r$) *are the eigenvalues of* *x* *and the expression* $\lambda_{1}e_{1}+\cdots\lambda _{r}e_{r}$ *is the spectral decomposition of* *x*.

Let $\lambda_{i}(x)$ ($i=1,2,\ldots,r$) denote the eigenvalues of *x*. In the rest of this paper, we write

$$\begin{aligned} \tau(x):=\max_{1\leq i\leq r}\lambda_{i}(x). \end{aligned}$$

Secondly, we give some definitions about a second-order cone complementarity function.

### Definition 2.1

([4])

If the mapping $\phi:R^{n}\times R^{n}\rightarrow R^{n}$ such that

$$\begin{aligned} \langle x,y\rangle=0,\quad x\in\mathcal{K}^{n}, y\in\mathcal {K}^{n}\quad \Leftrightarrow\quad \phi(x,y)=0, \end{aligned}$$

we say that the mapping *ϕ* is a second-order cone complementarity function on $\mathcal{K}^{n}$.

There are two well-known second-order cone complementarity functions. One is a Fischer–Burimister (FB) second-order cone complementarity function, that is, $\phi_{\mathrm{FB}}:R^{n}\times R^{n}\rightarrow R^{n}$,

$$\begin{aligned} \phi_{\mathrm{FB}}(x,y):=x+y-\sqrt{x^{2}+y^{2}}. \end{aligned}$$

Another is a natural residual function $\phi_{\mathrm{NR}}: R^{n}\times R^{n}\rightarrow R^{n}$,

$$\begin{aligned} \phi_{\mathrm{NR}}(x,y):=x-(x-y)_{+}, \end{aligned}$$

where $[x]_{+}$ denotes the metric projection of *x* onto $\mathcal {K}$. And by [14],

2.1 $$\begin{aligned} (2-\sqrt{2}) \bigl\Vert \phi_{\mathrm{NR}}(x,y) \bigr\Vert \leq \bigl\Vert \phi_{\mathrm{FB}}(x,y) \bigr\Vert \leq(2+\sqrt{2}) \bigl\Vert \phi_{\mathrm{NR}}(x,y) \bigr\Vert . \end{aligned}$$

In this paper, we consider a Fischer–Burimister(FB) second-order cone complementarity function, taking advantage of the knowledge about Jordan algebra, $\phi_{\mathrm{FB}}(x,y)$ can be also written as

$$\begin{aligned} \phi_{\mathrm{FB}}(x,y)=x+y- \bigl(\sqrt{\lambda_{1}}u^{1}+ \sqrt{\lambda_{2}}u^{2} \bigr). \end{aligned}$$

Here, $\{\lambda_{1}, \lambda_{2}\}$ and $\{u^{1},u^{2}\}$ such that

$$\begin{aligned}& \lambda_{i}:= \Vert x \Vert ^{2}+ \Vert y \Vert ^{2}+2(-1)^{i} \Vert x_{1}x_{2}+y_{1}y_{2} \Vert ,\quad i=1,2, \\& u^{i}:= \textstyle\begin{cases} \frac{1}{2} (1,(-1)^{i}\frac{x_{1}x_{2}+y_{1}y_{2}}{ \Vert x_{1}x_{2}+y_{1}y_{2} \Vert } )& \text{if }x_{1}x_{2}+y_{1}y_{2}\neq0,\\ \frac{1}{2} (1,(-1)^{i}\varpi )& \text{if }x_{1}x_{2}+y_{1}y_{2}=0, i=1,2, \end{cases}\displaystyle \end{aligned}$$

where $\varpi\in R^{n-1}$ is a vector satisfying $\|\varpi\|=1$.

### Definition 2.2

([4])

If the mapping $\Phi:R^{n}\times R^{n}\rightarrow R_{+}$ such that

$$\begin{aligned} \langle x,y\rangle=0,\quad x\in\mathcal{K}^{n},y\in\mathcal {K}^{n}\quad \Leftrightarrow\quad \Phi(x,y)=0, \end{aligned}$$

we call that the mapping Φ is a second-order cone merit function on $\mathcal{K}^{n}$.

In fact, we have $\Phi(x,y)=\|\phi(x,y)\|^{2}$ is a second-order cone merit function.

Let $\Phi_{\mathrm{FB}} (x,y):=\|\phi_{\mathrm{FB}}(x,y)\|^{2}$ represent a second-order cone merit function. Note that the second-order cone function $\phi _{\mathrm{FB}}$ is only semi-smooth, not continuously differentiable. But its corresponding second-order cone merit function is continuously differentiable.

Based on the definition of $R_{0}^{\omega}$-function in [14], we present the definition of stochastic *τ*-$R_{0}$ function, which will be used in the proof of boundedness of level sets.

### Definition 2.3

([20])

A function $F:R^{n}\rightarrow R^{n}$ is called a *τ*-$R_{0}$ function if for every infinite sequence $\{x^{k}\}\subseteq R^{n}$ that satisfies

$$\begin{aligned} \lim_{k\rightarrow\infty} \bigl\Vert x^{k} \bigr\Vert =\infty, \qquad\limsup_{k\rightarrow \infty}\tau \bigl( \bigl(-x^{k} \bigr)_{+} \bigr)< \infty,\qquad\limsup_{k\rightarrow\infty}\tau \bigl( \bigl(-F \bigl(x^{k} \bigr) \bigr)_{+} \bigr)< \infty, \end{aligned}$$

then $\limsup_{k\rightarrow\infty}\tau(x^{k}\sqcap f(x^{k}))=\infty$. Here, $x^{k}\sqcap f(x^{k})=x^{k}-(x^{k}-f(x^{k}))_{+}$, which is equivalent to $\phi_{\mathrm{NR}}(x^{k},f(x^{k}))$.

### Definition 2.4

A function $G:R^{n}\times\Omega\rightarrow R^{n}$ is called a stochastic *τ*-$R_{0}$ function on $R^{n}$ if for every infinite sequence $\{x^{k}\}\subseteq R^{n}$ that satisfies

$$\begin{aligned} \lim_{k\rightarrow\infty} \bigl\Vert x^{k} \bigr\Vert =\infty, \qquad\limsup_{k\rightarrow \infty}\tau \bigl( \bigl(-x^{k} \bigr)_{+} \bigr)< \infty,\qquad\limsup_{k\rightarrow\infty}\tau \bigl( \bigl(-G \bigl(x^{k},\omega \bigr) \bigr)_{+} \bigr)< \infty\quad \mbox{a.e.}, \end{aligned}$$

then $\mathcal{P}(\omega:\limsup_{k\rightarrow\infty}\tau(x^{k}\sqcap G(x^{k},\omega))=\infty)>0$.

Similar to the definition of equi-coerciveness in [20] , we give the definition of *τ*-equi-coerciveness.

### Definition 2.5

A function $H:R^{n}\times\Omega\rightarrow R^{n}$ is called *τ*-equi-coercive on $R^{n}$ if, for any $\{x^{k}\}\subseteq R^{n}$ satisfying $\|x^{k}\|\rightarrow\infty$, the existence of $\{\omega ^{k}\}\subseteq \operatorname{supp}\Omega$ with $\lim_{k\rightarrow\infty}\lambda _{i}(H(x^{k},\omega^{k}))=\infty$ ($\lim_{k\rightarrow\infty }\lambda_{i}((-H(x^{k},\omega^{k}))_{+})=\infty $) for some $i\in (1,\ldots,n)$ implies that

$$\begin{aligned} \mathcal{P} \Bigl(\omega: \lim_{k\rightarrow\infty}\lambda_{i} \bigl(H \bigl(x^{k},\omega \bigr) \bigr)=\infty \Bigr)>0 \quad \Bigl( \mathcal{P} \Bigl(\omega: \lim_{k\rightarrow\infty}\lambda _{i} \bigl( \bigl(-H \bigl(x^{k},\omega \bigr) \bigr)_{+} \bigr)= \infty \Bigr)>0 \Bigr). \end{aligned}$$

## CVaR model and its approximation problems

Before giving a CVaR model, we give the definition of Value-at-Risk (VaR). For a variable *x* and a parameter $u\in R_{+}$, the risk value less than level *u*, its probability is defined as

$$\begin{aligned} \mathcal{P} \bigl(g(x,\omega)\leq u \bigr)= \int_{g(x,\omega)\leq u}\rho(\omega )\,d\omega, \end{aligned}$$

where $\rho(\omega)$ denotes the probability density function of *ω*. For a given confidence level *α*, the definition of $\operatorname{VaR}_{\alpha}(x)$ about *x* is given as follows:

$$\begin{aligned} \operatorname{VaR}_{\alpha}(x):=\min \bigl\{ u\in R| \mathcal{P} \bigl(g(x,\omega)\leq u \bigr)\geq \alpha \bigr\} . \end{aligned}$$

Here, $g(x,\omega):R^{n}\times\Omega\rightarrow R$ denotes the loss function. Note that VaR is a popular risk measure, but it has undesirable properties such as a lack of subadditivity, convexity and fails to be coherent in the sense. However, CVaR can cover the shortage of VaR, that is, CVaR satisfies coherence and convexity. Besides, in references [17, 18], Rockafellar and Uryasev indicate that when minimizing CVaR, VaR can be obtained as the result of CVaR’s auxiliary.

For a given confidence level *α*, CVaR is defined as the conditional expectation of the loss associated with *x* relative to that loss being $\mathrm{VaR}_{\alpha}$ or greater, that is,

$$\begin{aligned} \operatorname{CVaR}_{\alpha}(x):=(1-\alpha)^{-1} \int_{g(x,\omega)\geq\operatorname{VaR}_{\alpha}(x)}g(x,\omega)\rho(\omega)\,d\omega. \end{aligned}$$

So, the model of minimizing the condition Value-at-Risk can be denoted by

3.1 $$\begin{aligned} \min \operatorname{CVaR}_{\alpha}(x). \end{aligned}$$

Set $G_{\alpha}(x,u):R^{n}\times R\rightarrow R$ is given by

$$\begin{aligned} G_{\alpha}(x,u)&=u+\frac{1}{1-\alpha} \int \bigl[g(x,\omega)-u \bigr]_{+}\rho (\omega)\,d\omega \\ &=u+(1-\alpha)^{-1}{\mathbf{E}} \bigl[ \bigl[g(x,\omega)-u \bigr]_{+} \bigr], \end{aligned} $$

where **E** denotes mathematics expectation. From references [17, 18], we can obtain minimizing $\mathrm{CVaR}_{\alpha}$(x) is equivalent to the minimizing function $G_{\alpha }(x,u)$. Therefore, CVaR model (3.1) is equivalent to the following optimization problem:

3.2 $$\begin{aligned} \min_{(x,u)\in(R^{n}\times R_{+})}G_{\alpha}(x,u). \end{aligned}$$

In this paper, we employ the second-order cone merit function $\Phi _{\mathrm{FB}}(x,f(x,\omega))$ to define the loss function in (3.2), and give the CVaR model for solving SSOCCP as follows:

3.3 $$\begin{aligned} \min_{(x,u)\in(R^{n}\times R_{+})}\Theta(x,u):=u+(1-\alpha )^{-1}{\mathbf{E}} \bigl[ \bigl[\Phi_{\mathrm{FB}} \bigl(x,f(x,\omega) \bigr)-u \bigr]_{+} \bigr]. \end{aligned}$$

Here, $\alpha\in(0,1)$ denotes a given confidence level.

One main difficulty in dealing with (3.3) is that the model contains an expectation, which is difficult to calculate in general. So, in this paper, we employ the sample average approximation method to approximate the expectation.

The sample average approximation method uses the sample average value approximates the expected [16]. That is, for an integrable function $\psi:\Omega\rightarrow R$, ${\mathbf{E}}[\psi(\omega)]\approx \frac{1}{N_{k}}\sum_{\omega^{i}\in\Omega_{k}}\psi(\omega^{i})$, where $\Omega_{k}:=\{\omega^{1},\ldots,\omega^{N_{k}}\}\subseteq\Omega$ and $\omega^{i}$ ($i=1,2,\ldots,N_{k}$) is an independently and identically distributed sample. We assume that $\{N_{k}\}$ tends to infinity as *k* increases. The strong law of large numbers guarantees that this procedure converges with probability one (abbreviated by “w.p.1.”), that is,

3.4 $$\begin{aligned} \lim_{k\rightarrow\infty}\frac{1}{N_{k}}\sum _{\omega^{i}\in\Omega _{k}}\psi \bigl(\omega^{i} \bigr)={\mathbf{E}} \bigl[ \psi(\omega) \bigr],\quad \mbox{w.p.1}. \end{aligned}$$

By employing the sample average approximate method, we can obtain the approximates problem of CVaR model as follows:

$$\begin{aligned} \min_{(x,u)\in(R^{n}\times R_{+})}\Theta^{k}(x,u):=u+(1- \alpha )^{-1}\frac{1}{N_{k}}\sum_{\omega^{i}\in\Omega_{k}} \bigl[\Phi_{\mathrm{FB}} \bigl(x,f(x,\omega) \bigr)-u \bigr]_{+}, \end{aligned}$$

where $\Omega_{k}=\{\omega^{1},\ldots,\omega^{N_{k}}\}$. And $N_{k}$ satisfies $N_{k}\rightarrow\infty$ as $k\rightarrow\infty$.

The other difficulty is that $[\cdot]_{+}$ is not differentiable everywhere. So the objective function of CVaR model is nonsmooth, which makes us apply general optimization algorithm unable to solve this problem. Therefore, we use the smoothing function presented by Li in [13] to smooth $[\cdot]_{+}$. The smoothing form of the function $[t]_{+} $ is given as follows:

$$\begin{aligned} h_{\mu}(t)=\mu\ln \bigl(\exp^{\frac{t}{\mu}}+1 \bigr). \end{aligned}$$

In reference [13], Li et al. prove that the following formulations hold:

3.5 $$\begin{aligned} \lim_{\mu\rightarrow0}h_{\mu}(t)=[t]_{+} \quad\mbox{and} \quad0\leq h_{\mu}(t)-[t]_{+}\leq\mu\ln2. \end{aligned}$$

For simplicity, let

$$\begin{aligned} h(x,u,\omega):= \bigl[\Phi_{\mathrm{FB}} \bigl(x,f(x,\omega) \bigr)-u \bigr]_{+}. \end{aligned}$$

Then, for any $\mu>0$, we use

$$\begin{aligned} h_{\mu}(x,u,\omega):=\mu\ln \bigl(\exp^{\frac{\Phi_{\mathrm{FB}}(x,f(x,\omega ))-u}{\mu}}+1 \bigr) \end{aligned}$$

to denote the smoothing function of $h(x,u,\omega)$. It is easy to get that

$$\begin{aligned} \lim_{\mu\rightarrow0}h_{\mu}(x,u,\omega)=h(x,u,\omega). \end{aligned}$$

Especially, following from (3.5), the smoothing function $h_{\mu}(x,u,\omega)$ satisfies

3.6 $$\begin{aligned} 0\leq h_{\mu}(x,u,\omega)-h(x,u,\omega)\leq\mu\ln2. \end{aligned}$$

We then combine the sample average approximation method and the smoothing method together, to construct a smoothing sample average approximation problem of the CVaR model as follows:

3.7 $$\begin{aligned} \min_{(x,u)\in(R^{n}\times R_{+})} \Theta^{k}_{\mu}(x,u):=u+(1- \alpha )^{-1}\frac{1}{N_{k}}\cdot\sum_{\omega^{i}\in\Omega_{k}} \mu\ln \bigl(\exp^{\frac {\Phi_{\mathrm{FB}}(x,f(x,\omega))-u}{\mu}}+1 \bigr). \end{aligned}$$

## The boundedness of level set

The boundedness of level set is important since it ensures that the solutions of the optimization problem (3.3) are bounded. So, we consider that when $f(x,\omega)$ is a stochastic *τ*-$R_{0}$ function and *τ*-equi-coercive, the level set of $\Theta(x,u)$ is bounded.

### Theorem 4.1

*Suppose that there exists* $\overline{\omega}\in \operatorname{supp}\Omega$ *such that* $f(x,\overline{\omega})$ *is a* *τ*-$R_{0}$ *function on* $R^{n}$, *and* $f(x,\omega)$ *is* *τ*-*equi*-*coercive on* $R^{n}$. *Then* $f(x,\omega)$ *is a stochastic* *τ*-$R_{0}$ *function for every* $\omega\in\Omega$.

### Proof

Suppose that $\{x^{k}\}\subseteq R^{n}$ satisfies

$$\begin{aligned} \lim_{k\rightarrow\infty} \bigl\Vert x^{k} \bigr\Vert =\infty, \qquad\limsup_{k\rightarrow \infty}\tau \bigl( \bigl(-x^{k} \bigr)_{+} \bigr)< \infty,\qquad\limsup_{k\rightarrow\infty}\tau \bigl( \bigl(-f \bigl(x^{k},\omega \bigr) \bigr)_{+} \bigr)< \infty\quad \mbox{a.e.} \end{aligned}$$

If $\limsup_{k\rightarrow\infty}\tau((-f(x^{k},\overline{\omega }))_{+})=\infty$, then at least there exists one $i\in(1,\ldots,n)$ and $\{x^{k_{i}}\}\subseteq\{x^{k}\}$ such that $\lim_{k_{i}\rightarrow \infty}\lambda_{i}((-f_{i}(x^{k_{i}},\overline{\omega}))_{+})=\infty$. Since $f(x,\omega)$ is *τ*-equi-coercive, then we have

$$\begin{aligned} \mathcal{P} \Bigl(\omega: \lim_{k_{i}\rightarrow\infty}\lambda _{i} \bigl( \bigl(-f \bigl(x^{k_{i}},\omega \bigr) \bigr)_{+} \bigr)= \infty \Bigr)>0, \end{aligned}$$

which contradicts

$$\begin{aligned} \limsup_{k\rightarrow\infty}\tau \bigl( \bigl(-f \bigl(x^{k}, \omega \bigr) \bigr)_{+} \bigr)< \infty\quad \mbox{a.e.} \end{aligned}$$

Hence, we must have

$$\begin{aligned} \limsup_{k\rightarrow\infty}\tau \bigl( \bigl(-f \bigl(x^{k}, \overline{\omega } \bigr) \bigr)_{+} \bigr)< \infty. \end{aligned}$$

Since $f(x,\overline{\omega})$ is a *τ*-$R_{0}$ function, $\limsup_{k\rightarrow\infty}\tau(x^{k}\sqcap f(x^{k},\overline{\omega}))=\infty $. Using $f(x,\omega)$ is *τ*-equi-coercive again, we have

$$\begin{aligned} \mathcal{P} \Bigl(\omega: \limsup_{k\rightarrow\infty}\tau \bigl(x^{k}\sqcap f \bigl(x^{k},\omega \bigr) \bigr)=\infty \Bigr)>0. \end{aligned}$$

Therefore, $f(x,\omega)$ is a stochastic *τ*-$R_{0}$ function on $R^{n}$. □

### Theorem 4.2

*Suppose that there exists* $\overline{\omega}\in \operatorname{supp}\Omega$ *such that* $\rho(\overline{\omega})>0$ *and* $f(x,\overline{\omega})$ *is a* *τ*-$R_{0}$ *function*, $f(x,\omega)$ *is* *τ*-*equi*-*coercive*. *Then* $L_{\gamma}^{\Theta}(x,u)=\{(x,u)|\Theta(x,u)\leq\gamma\}$ *is bounded*.

### Proof

Suppose that $L_{\gamma}^{\Theta}(x,u)$ is unbounded, there exists a sequence $\{(x^{k},u^{k})\}\subset L_{\gamma}^{\Theta}(x,u)$ such that $\lim_{k\rightarrow\infty}\|x^{k} \|=\infty$ or $\lim_{k\rightarrow \infty}|u^{k}|=\infty$. By (3.3) and the boundedness of $L_{\gamma}^{\Theta}(x,u)$, we can get that $\lim_{k\rightarrow\infty }|u^{k}|=\infty$ cannot hold. So, there exists a sequence $\{x^{k}\}$ satisfying $\lim_{k\rightarrow\infty}\|x^{k}\|=\infty$.

Firstly, we prove

4.1 $$\begin{aligned} \lim_{k\rightarrow\infty}\Phi_{\mathrm{FB}} \bigl(x^{k},f \bigl(x^{k},\omega \bigr) \bigr)=\infty. \end{aligned}$$

On the contrary, for an unbounded sequence $\{x^{k}\}$, there exists $\delta>0$ such that $\omega\in\overline{B}:=\{\omega\in\Omega|\|\omega -\overline{\omega}\|\leq\delta\}$, by Theorem 4.1, $f(x,\omega)$ is a stochastic *τ*-$R_{0}$ function. If $\limsup_{k\rightarrow\infty }\tau((-x^{k})_{+})=\infty$, then (through a subsequence) $\| (-x^{k})_{+}\|\rightarrow\infty$. Note that

4.2 $$\begin{aligned} \begin{aligned}[b] &\Phi_{\mathrm{FB}} \bigl(x^{k},f \bigl(x^{k},\omega \bigr) \bigr) \\ &\quad= \bigl\Vert \phi_{\mathrm{FB}} \bigl(x^{k},f \bigl(x^{k},\omega \bigr) \bigr) \bigr\Vert ^{2} \\ &\quad\geq \bigl\Vert \bigl[\phi_{\mathrm{FB}} \bigl(x^{k},f \bigl(x^{k},\omega \bigr) \bigr) \bigr]_{+} \bigr\Vert ^{2} \\ &\quad\geq\frac{1}{2} \bigl( \bigl\Vert \bigl(-x^{k} \bigr)_{+} \bigr\Vert ^{2}+ \bigl\Vert \bigl(-f \bigl(x^{k},\omega \bigr) \bigr)_{+} \bigr\Vert ^{2} \bigr), \end{aligned} \end{aligned}$$

where the first inequality is due to the nonexpansiveness of the projection and $[0]_{+}=0$, the second inequality follows from Lemma 5.2 in [15]. By (4.2), we have $\lim_{k\rightarrow\infty }\Phi_{\mathrm{FB}}(x^{k}, f(x^{k}, \omega))=\infty$. This contradicts the boundedness of $\Phi_{\mathrm{FB}}(x^{k},f(x^{k},\omega))$. If $\limsup_{k\rightarrow\infty}\tau((-f(x^{k}, \omega))_{+})=\infty$, by (4.2), we get the same contradiction. So, we have

$$\begin{aligned} \limsup_{k\rightarrow\infty}\tau \bigl( \bigl(-x^{k} \bigr)_{+} \bigr)< \infty\quad \mbox{and}\quad \limsup _{k\rightarrow\infty}\tau \bigl( \bigl(-f \bigl(x^{k},\omega \bigr) \bigr)_{+} \bigr)< \infty. \end{aligned}$$

That is, the unbounded sequence $\{x^{k}\}$ satisfies the conditions in Definition 2.4. By Definition 2.4, we have $\mathcal{P}(\omega\in\Omega: \limsup_{k\rightarrow\infty}\tau (x^{k}\sqcap f(x^{k},\omega))=\infty)>0$. So, $x^{k}\sqcap f(x^{k},\omega)\rightarrow\infty$, that is, $\phi _{\mathrm{NR}}(x^{k},f(x^{k},\omega))\rightarrow\infty$. Taking formulation (2.1) into account, we have $\|\phi_{\mathrm{FB}}(x^{k}, f(x^{k},\omega ))\|\rightarrow\infty$. This contradicts the boundedness of $\Phi _{\mathrm{FB}}(x^{k},f(x^{k},\omega))$. So $\lim_{k\rightarrow\infty}\Phi _{\mathrm{FB}}(x^{k}, f(x^{k},\omega))=\infty$.

Secondly, because of the continuity of *ρ*, there exists a constant $\overline{\rho}>0$, and $\rho(\omega)\geq\overline{\rho}$. So, for every *k*, there exists $\omega^{k}\in\overline{B}$ such that

4.3 $$\begin{aligned} \bigl\Vert \Phi_{\mathrm{FB}} \bigl(x^{k},f \bigl(x^{k}, \omega^{k} \bigr) \bigr) \bigr\Vert =\min_{\omega\in\overline{B}} \bigl\Vert \Phi_{\mathrm{FB}} \bigl(x^{k},f \bigl(x^{k}, \omega \bigr) \bigr) \bigr\Vert . \end{aligned}$$

By the fact that $\int_{\overline{B}}\,d\omega>0$ and formulation (4.1), we have that

$$\begin{aligned} \Theta \bigl(x^{k},u \bigr)&\geq \int_{\overline{B}}u+(1-\alpha)^{-1} \bigl( \bigl(\Phi _{\mathrm{FB}} \bigl(x^{k},f \bigl(x^{k},\omega \bigr) \bigr)-u \bigr)_{+} \bigr)\rho(\omega)\,d\omega \\ & \geq u+(1-\alpha)^{-1} \bigl( \bigl(\Phi_{\mathrm{FB}} \bigl(x^{k},f \bigl(x^{k},\omega ^{k} \bigr) \bigr)-u \bigr)_{+} \bigr)\overline{\rho} \int_{\overline{B}}\,d\omega \\ &\rightarrow\infty\quad(k\rightarrow\infty). \end{aligned}$$

This contradicts the boundedness of $L_{\gamma}^{\Theta}(x,u)$. So we can get $L_{\gamma}^{\Theta}(x,u)=\{(x,u)|\Theta(x,u)\leq\gamma\}$ is bounded. □

We denote $S^{*}$ and $S_{k}^{*}$ as the sets of optimal solutions of problems (3.3) and (3.7), respectively.

### Theorem 4.3

*Suppose that there exists* $\overline{\omega}\in \operatorname{supp}\Omega$ *such that* $f(x,\overline{\omega})$ *is a* *τ*-$R_{0}$ *function*, *and* $f(x, \omega )$ *is* *τ*-*equi*-*coercive on* $R^{n}$. *Then* $\lim_{x\rightarrow\infty }\Theta_{\mu}^{k}(x,u)=+\infty$ *when* *k* *is large enough*. *In particular*, $S_{k}^{*}$ *is nonempty and bounded for every* *k* *sufficiently large*.

### Proof

By Theorem 4.1, for any $\omega\in\Omega$, $f(x,\omega)$ is a stochastic *τ*-$R_{0}$ function. Thus, in the similar way to the proof of Theorem 4.2, we can get $\lim_{x\rightarrow\infty}\Theta _{\mu}^{k}(x,u)=+\infty$. □

## Analysis convergence

We next study the convergence of the smoothing sample average approximation problem.

### Theorem 5.1

*Suppose that for any* *k*, $(x^{k},u^{k})$ *is a global optimal solution of problem* (3.7), $(\bar{x},\bar{u})$ *is an accumulation point of sequence* $\{(x^{k},u^{k})\}$. *Then* $(\bar {x},\bar{u})$ *is a globally optimal solution of CVaR model* (3.3) *which holds with probability one*.

### Proof

Without loss of generality, we assume that $\lim_{k\rightarrow\infty }(x^{k},u^{k})=(\bar{x},\bar{u})$. Let B be a compact set including the whole sequence $\{(x^{k},u^{k})\}$. $Z\subseteq R^{+}$ is a compact set including $\{\mu^{k}\}$. Because $h_{\mu}(x,u,\omega)$ is a continuously differentiable function on the compact set $B\times\Omega \times Z$, we can obtain that there exists a constant $C>0$ such that, for any $(x,u,\omega)\in B\times\Omega$ and $\mu\in Z$, the following formulation holds:

5.1 $$\begin{aligned} \bigl\Vert \nabla h_{\mu}(x,u,\omega) \bigr\Vert \le C. \end{aligned}$$

Besides, from mean value theorem, for each $x^{k}$, $u^{k}$, $\omega^{i}$, and $\mu^{k}$, there exists $\alpha_{ki}\in(0,1)$ such that $(x^{ki},u^{ki})=\alpha_{ki}(x^{k},u^{k})+(1-\alpha_{ki}) (\bar{x},\bar{u})\in B$, we then have

5.2 $$ \begin{aligned}[b] &h_{\mu^{k}} \bigl(x^{k}, u^{k},\omega^{i} \bigr)-h_{\mu^{k}} \bigl(\bar{x},\bar{u},\omega^{i} \bigr) \\ &\quad=\nabla h_{\mu^{k}} \bigl(x^{ki},u^{ki}, \omega^{i} \bigr)^{T} \bigl( \bigl(x^{k},u^{k} \bigr)-(\bar{x},\bar{u}) \bigr). \end{aligned} $$

Therefore, from (5.1) and (5.2), we have

5.3 $$ \begin{aligned}[b] & \bigl\vert \Theta^{k}_{\mu^{k}} \bigl(x^{k},u^{k} \bigr)- \Theta^{k}_{\mu^{k}}( \bar{x},\bar {u}) \bigr\vert \\ &\quad\leq \bigl\vert u^{k}-\bar{u} \bigr\vert +(1- \alpha)^{-1}\frac{1}{N_{k}}\sum_{\omega^{i}\in \Omega_{k}} \bigl\vert h_{\mu^{k}} \bigl(x^{k},u^{k}, \omega^{i} \bigr)-h_{\mu^{k}} \bigl(\bar{x},\bar {u}, \omega^{i} \bigr) \bigr\vert \\ &\quad\leq \bigl\vert u^{k}-\bar{u} \bigr\vert +(1- \alpha)^{-1}\frac{1}{N_{k}}\sum_{\omega^{i}\in \Omega_{k}} \bigl\Vert \nabla h_{\mu^{k}} \bigl(x^{ki},u^{ki}, \omega^{i} \bigr) \bigr\Vert \\ &\qquad{}\cdot \bigl\Vert \bigl(x^{k},u^{k} \bigr)-( \bar{x},\bar{u}) \bigr\Vert \\ &\quad\leq \bigl\vert u^{k}-\bar{u} \bigr\vert +\frac{C}{(1-\alpha)} \cdot\frac{1}{N_{k}}\sum_{\omega^{i}\in \Omega_{k}} \bigl\Vert \bigl(x^{k},u^{k} \bigr)-(\bar{x},\bar{u}) \bigr\Vert \\ &\quad\xrightarrow{k\rightarrow\infty}0,\quad \mbox{w.p.1}. \end{aligned} $$

On the other hand, from (3.4) and (3.6), we have

5.4 $$ \begin{aligned}[b] & \bigl\vert \Theta^{k}_{\mu^{k}}( \bar{x},\bar{u})-\Theta(\bar{x},\bar{u}) \bigr\vert \\ &\quad\leq(1-\alpha)^{-1} \biggl\vert \frac{1}{N_{k}}\sum _{\omega^{i}\in \Omega_{k}}h_{\mu^{k}} \bigl(\bar{x},\bar{u}, \omega^{i} \bigr)-{\mathbf{E}} \bigl[h(\bar{x},\bar {u},\omega) \bigr] \biggr\vert \\ &\quad\leq(1-\alpha)^{-1} \biggl[\frac{1}{N_{k}}\sum _{\omega^{i}\in \Omega_{k}} \bigl\vert h_{\mu^{k}} \bigl(\bar{x},\bar{u}, \omega^{i} \bigr)-h \bigl(\bar{x},\bar {u},\omega^{i} \bigr) \bigr\vert \\ &\qquad{}+ \biggl\vert \frac{1}{N_{k}}\sum_{\omega^{i}\in \Omega_{k}}h \bigl(\bar{x},\bar{u},\omega^{i} \bigr)-{\mathbf{E}} \bigl[h(\bar{x}, \bar {u},\omega) \bigr] \biggr\vert \biggr] \\ &\quad\leq(1-\alpha)^{-1} \biggl[\frac{1}{N_{k}}\sum _{\omega^{i}\in \Omega_{k}}\mu_{k}\ln2 \\ &\qquad{}+ \biggl\vert \frac{1}{N_{k}}\sum_{\omega^{i}\in \Omega_{k}}h \bigl(\bar{x},\bar{u},\omega^{i} \bigr)-{\mathbf{E}} \bigl[h(\bar{x}, \bar {u},\omega) \bigr] \biggr\vert \biggr] \\ &\quad\xrightarrow{k\rightarrow\infty}0, \quad \mbox{w.p.1}. \end{aligned} $$

Similarly, we obtain

5.5 $$\begin{aligned} \lim_{k\rightarrow\infty}\Theta^{k}_{\mu^{k}}(x,u)= \Theta(x,u),\quad \mbox{w.p.1}. \end{aligned}$$

Since

$$\begin{aligned} & \bigl\vert \Theta^{k}_{\mu^{k}} \bigl(x^{k},u^{k} \bigr)-\Theta(\bar{x},\bar{u}) \bigr\vert \\ &\quad\leq \bigl\vert \Theta^{k}_{\mu^{k}} \bigl(x^{k},u^{k} \bigr)-\Theta^{k}_{\mu^{k}}( \bar {x},\bar{u}) \bigr\vert + \bigl\vert \Theta^{k}_{\mu^{k}}( \bar{x},\bar{u})-\Theta(\bar{x},\bar{u}) \bigr\vert , \end{aligned}$$

from (5.3) and (5.4) we have

5.6 $$\begin{aligned} \lim_{k\rightarrow\infty}\Theta^{k}_{\mu^{k}} \bigl(x^{k},u^{k} \bigr)=\Theta(\bar {x},\bar{u}),\quad \mbox{w.p.1}. \end{aligned}$$

In fact, for each *k*, due to $(x^{k},u^{k})$ being a global optimal solution of problem (3.7), we have

5.7 $$\begin{aligned} \Theta^{k}_{\mu^{k}} \bigl(x^{k},u^{k} \bigr)\leq\Theta^{k}_{\mu^{k}}(x,u),\quad\forall (x,u)\in R^{n}\times R_{+}. \end{aligned}$$

Taking limits to (5.7), by (5.5) and (5.6), we have

$$\begin{aligned} \Theta(\bar{x},\bar{u})\leq\Theta(x,u),\quad \mbox{w.p.1}, \forall (x,u)\in R^{n}\times R_{+}. \end{aligned}$$

 □

Note that approximation problem (3.7) is a nonconvex programming problem, so when we solve this problem, we will obtain stationary points rather than global optimal solutions. Therefore, we will analyze the convergence of problem (3.3)’s and (3.7)’s stationary points. Firstly, we give the definition of stationary points of (3.3) and (3.7), respectively.

### Definition 5.1

([7])

The Clarke generalized gradient of $f(x)$ with respect to *x* is defined as

$$\begin{aligned} \partial_{x}f(x):=\operatorname{conv} \Bigl\{ \lim _{y\rightarrow x,y\in D_{f(\cdot)}}\nabla_{x}f(y) \Bigr\} , \end{aligned}$$

where $D_{f(\cdot)}$ denotes the set of points near *x* where the function $f:R^{n}\rightarrow R$ near *x* is Frechét differentiable, $\nabla_{x}f(y)$ denotes $f(y)$ general gradient with respect to x, and conv denotes convex hull of a set.

### Definition 5.2

Let $d^{ki}_{\mu^{k}}(x^{k},u^{k},\omega^{i}):= \exp^{\frac{\Phi_{\mathrm{FB}}(x^{k},f(x^{k},\omega^{i}))-u^{k}}{\mu^{k}}}$, for each fixed *k*, if $(x^{k},u^{k})$ satisfies

5.8 $$\begin{aligned}& (1-\alpha)^{-1}\frac{1}{N_{k}}\sum_{\omega^{i}\in\Omega_{k}} \frac {d^{ki}_{\mu^{k}}(x^{k},u^{k},\omega^{i})}{ 1+d^{ki}_{\mu^{k}}(x^{k},u^{k},\omega^{i})}\cdot \bigl(\nabla\Phi _{\mathrm{FB}} \bigl(x^{k},f \bigl(x^{k},\omega^{i} \bigr) \bigr), \mathbf{0} \bigr)=0, \\& 1-(1-\alpha)^{-1}\frac{1}{N_{k}}\sum_{\omega^{i}\in\Omega_{k}} \frac {d^{ki}_{\mu^{k}}(x^{k},u^{k},\omega^{i})}{1+d^{ki}_{\mu ^{k}}(x^{k},u^{k},\omega^{i})}=0 , \end{aligned}$$

where $\mathbf{0}\in R^{l}$ is zero vector, we call $(x^{k},z^{k},u^{k})$ a stationary point of (3.7).

### Definition 5.3

If $(\bar{x},\bar{u})$ satisfies

5.9 $$\begin{aligned} 0\in\partial_{(x,u)}\Theta(\bar{x},\bar{u}), \end{aligned}$$

we call $(\bar{x},\bar{u})$ a stationary point of (3.3).

### Theorem 5.2

*Suppose that for any* *k*, $(x^{k},u^{k})$ *is a stationary point of problem* (3.7), $(\bar{x},\bar{u})$ *is an accumulation point of the sequence* $\{(x^{k},u^{k})\}$. *Then* $(\bar {x},\bar{u})$ *is a stationary point of CVaR model* (3.3) *which holds with probability one*.

### Proof

Without loss of generality, we assume that

$$\begin{aligned} \lim_{k\rightarrow\infty} \bigl(x^{k},u^{k} \bigr)=( \bar{x},\bar{u}). \end{aligned}$$

Let $\textbf{B}:=\{x|||x-\overline{x}||\leq1\}$, $\textbf{C}:=\{u||u-\overline{u}|\leq1\}$. Because $\Phi _{\mathrm{FB}}(x,f(x,\omega))$ is a continuously differentiable function on the compact set $\textbf{B}\times\Omega$, we can obtain that there exists a constant $D>0$ such that, for any $(x,\omega)\in\textbf{B}\times \Omega$, the following formulation holds:

5.10 $$\begin{aligned} \bigl\Vert \nabla\Phi_{\mathrm{FB}} \bigl(x,f(x,\omega) \bigr) \bigr\Vert \le D. \end{aligned}$$

From mean value theorem (Theorem 2.19 in [9]), we obtain that there exists $\beta\in(0,1)$ such that $(\tilde{x},\tilde{y})=\beta (x',y')+ (1-\beta)(x',y')\in\textbf{B} $ and

5.11 $$ \begin{aligned}[b] & \bigl\vert \Phi_{\mathrm{FB}} \bigl(x',y' \bigr)-\Phi_{\mathrm{FB}} \bigl(x'',y'' \bigr) \bigr\vert \\ &\quad= \bigl\vert \nabla\Phi_{\mathrm{FB}}(\tilde{x},\tilde{y})^{T} \bigl( \bigl(x',y' \bigr)- \bigl(x'',y'' \bigr) \bigr) \bigr\vert \\ &\quad\leq D \bigl\Vert \bigl(x',y' \bigr)- \bigl(x'',y'' \bigr) \bigr\Vert . \end{aligned} $$

Let

5.12 $$\begin{aligned} \vartheta(x,u,\omega):=u+(1-\alpha)^{-1} \bigl[\Phi_{\mathrm{FB}} \bigl(x,f(x,\omega) \bigr)-u \bigr]_{+}, \end{aligned}$$

we obtain $\Theta(x,u)={\mathbf{E}}[\vartheta(x,u,\omega)]$. From (5.10), (5.11), and the inequality $|[x]_{+}-[y]_{+}|\leq|x-y|$, we obtain that

$$\begin{aligned} \begin{aligned} & \bigl\vert \vartheta \bigl(x',u',\omega \bigr)-\vartheta \bigl(x'',u'',\omega \bigr) \bigr\vert \\ &\quad\leq \bigl\vert u'-u'' \bigr\vert +\frac{1}{1-\alpha} \bigl[\Phi_{\mathrm{FB}} \bigl(x',f \bigl(x',\omega \bigr) \bigr)-\Phi_{\mathrm{FB}} \bigl(x'',f \bigl(x'', \omega \bigr) \bigr)+ \bigl\vert u'-u'' \bigr\vert \bigr] \\ &\quad\leq \bigl\vert u'-u'' \bigr\vert +\frac{1}{1-\alpha} \bigl[D \bigl\Vert \bigl(x',y' \bigr)- \bigl(x'',y'' \bigr) \bigr\Vert + \bigl\vert u'-u'' \bigr\vert \bigr]. \end{aligned} \end{aligned}$$

Therefore, we get

$$\begin{aligned} {\mathbf{E}} \bigl[\partial_{(x,u)}\vartheta(\bar{x}, \bar{u},\omega) \bigr]\subseteq \partial_{(x,u)}\Theta(\bar{x},\bar{u}, \omega), \end{aligned}$$

by Theorem 9 in [19]. Therefore, in order to prove $0\in\partial_{(x,u)}\Theta(\bar{x},\bar {u},\omega)$, we only prove the following formulation holds:

5.13 0∈E[∂(x,u)ϑ(x¯,u¯,ω)]=E[(01)+(1−α)−1⋅r⋅((∇ΦFB(x¯,f(x¯,ω),0))−1)|(∇ΦFB(x¯,f(x¯,ω),0)−1r∈∂(x,u)[ΦFB(x¯,f(x¯,ω))−u¯]+],

where $\mathbf{0}\in R^{l}$ is a zero vector and

$$\begin{aligned} \partial_{(x,u)} \bigl[\Phi_{\mathrm{FB}} \bigl(\bar{x},f(x,\omega) \bigr)- \bar{u} \bigr]_{+}:= \textstyle\begin{cases} 1, & \Phi_{\mathrm{FB}}(\bar{x},f(\bar{x},\omega))-\bar{u}>0,\\ [0,1], & \Phi_{\mathrm{FB}}(\bar{x},f(\bar{x},\omega))-\bar{u}=0,\\ 0, & \Phi_{\mathrm{FB}}(\bar{x},f(\bar{x},\omega))-\bar{u}< 0. \end{cases}\displaystyle \end{aligned}$$

Next, we prove (5.13) holds. Let $d_{\mu}(x,u,\omega):=\exp [\frac{\Phi_{\mathrm{FB}}(x,f(x,\omega))-u}{\mu}]$ and

5.14 $$\begin{array}{rl} & \zeta_{\mu }(x,u,\omega )\\ & \;=\left\{ \begin{array}{cc} \left(\begin{array}{c} {\left(1-\alpha \right)}^{-1}\frac{d_{\mu }(x,u,\omega )}{1+d_{\mu }(x,u,\omega )}((\nabla \Phi_{\mathrm{FB}}(x,f(x,\omega )),0))\\ 1-{\left(1-\alpha \right)}^{-1}\frac{d_{\mu }(x,u,\omega )}{1+d_{\mu }(x,u,\omega )} \end{array}\right), & \mu \neq 0,\\ \left\{\left(\begin{array}{c} 0\\ 1 \end{array}\right)+{\left(1-\alpha \right)}^{-1}\cdot r\cdot \left(\begin{array}{c} \left(\nabla \Phi_{\mathrm{FB}}(x,f(x,\omega )),0\right)\\ -1 \end{array}\right)\right\}, & \mu =0, \end{array}\right. \end{array}$$

where $r\in\partial_{(x,u)}[\Phi_{\mathrm{FB}}(x,f(x,\omega))-u]_{+}$.

Next, we will prove that $\zeta_{\bar{\mu}}(x,u,\omega)$ is upper semicontinuous on $(\bar{x},\bar{u})$. When $\mu\neq0$, by (5.14), we know that $\zeta_{\bar{\mu}}(x,u,\omega)$ is a monotropic function near $(\bar{x},\bar{u})$. So, because $\Phi _{\mathrm{FB}}(x,f(x,\omega))$ is continuously differentiable about *x*, we get that $\zeta_{\bar{\mu}}(x,u,\omega)$ is continuous on $(\bar{x},\bar {u})$. When $\mu=0$, it is easy to find that $\zeta_{0}(x,u,\omega)$ is closed on $(\bar{x},\bar{u})$. Taking (5.10) into account, we have $\zeta_{0}(x,u,\omega)$ is uniform compact near $(\bar{x},\bar {u})$. Therefore, from Lemma 3.5 of [6], we know that $\zeta _{0}(x,u,\omega)$ is upper semicontinuous on $(\bar{x},\bar{u})$. Based on the above argument, $\zeta_{\bar{\mu}}(x,u,\omega)$ is upper semicontinuous on $(\bar{x},\bar{u})$.

Noting that Ω is a compact set and $\Phi_{\mathrm{FB}}(x,f(x,\omega))$ is continuously differentiable about *x*, we obtain that $\zeta_{\mu }(x,u,\omega)$ is a bounded and compact set value function in $\textbf {B}\times\textbf{C}\times\Omega$. So, the conditions in Lemma 3.4 [6] hold for the function $\zeta_{\mu}(x,u,\omega)$ since the formulation of Definition 5.2 is equivalent to

$$\begin{aligned} 0\in\frac{1}{N_{k}}\sum_{\omega^{i}\in\Omega _{k}} \zeta_{\mu^{k}} \bigl(x^{k},y^{k},z^{k},u^{k}, \omega \bigr). \end{aligned}$$

From Lemma 3.4 of [6], we get that formulation (5.9) holds. □

## Conclusions

In this paper, we consider a stochastic second-order cone complementarity problem. The boundedness of level sets of $\Theta(x,u)$ is important since it ensures that the solutions of the optimization problem (3.2) are bounded. Then, because for all $\omega\in \Omega$, SSOCCP (1.2) may have no solutions, we construct a reasonable deterministic model that is a conditional value-at-risk model and regard the solutions of this model as the solutions of SSOCCP. About the CVaR model, there may be two difficulties for solving the model. One is that the objective function is a non-smoothing function. The other is approximation of the expectation. We use the sample average approximate method and the smoothing method to solve these two difficulties. Moreover, we give convergence analysis of a global optimal solutions sequence and a stationary points sequence of smoothing sample average approximation problems.
